# Spatial lipidomics reveals zone-specific hepatic lipid alteration and remodeling in metabolic dysfunction-associated steatohepatitis

**DOI:** 10.1016/j.jlr.2024.100599

**Published:** 2024-07-18

**Authors:** Patcharamon Seubnooch, Matteo Montani, Jean-Francois Dufour, Mojgan Masoodi

**Affiliations:** 1 Institute of Clinical Chemistry, Inselspital, Bern University Hospital, Bern, Switzerland; 2 Graduate School for Cellular and Biomedical Sciences, University of Bern, Switzerland; 3 Institute of Tissue Medicine and Pathology, University of Bern, Bern, Switzerland; 4 Department for BioMedical Research, Visceral Surgery and Medicine, University of Bern, Bern, Switzerland

**Keywords:** liver lipid metabolism, liver zonation, lipid distribution, hepatic lipid zonation, steatotic liver disease, MASH, NASH, MASLD, NAFLD

## Abstract

Alteration in lipid metabolism plays a pivotal role in developing metabolic dysfunction-associated steatohepatitis (MASH). However, our understanding of alteration in lipid metabolism across liver zonation in MASH remains limited. Within this study, we investigated MASH-associated zone-specific lipid metabolism in a diet and chemical-induced MASH mouse model. Spatial lipidomics using mass spectrometry imaging in a MASH mouse model revealed 130 lipids from various classes altered across liver zonation and exhibited zone-specific lipid signatures in MASH. Triacylglycerols, diacylglycerols, sphingolipids and ceramides showed distinct zone-specific changes and re-distribution from pericentral to periportal localization in MASH. Saturated and monounsaturated fatty acids (FA) were the primary FA composition of increased lipids in MASH, while polyunsaturated FAs were the major FA composition of decreased lipids. We observed elevated fibrosis in the periportal region, which could be the result of observed metabolic alteration across zonation. Our study provides valuable insights into zone-specific hepatic lipid metabolism and demonstrates the significance of spatial lipidomics in understanding liver lipid metabolism. Identifying unique lipid distribution patterns may offer valuable insights into the pathophysiology of MASH and facilitate the discovery of diagnostic markers associated with liver zonation.

## Graphical abstract

**Figure:**
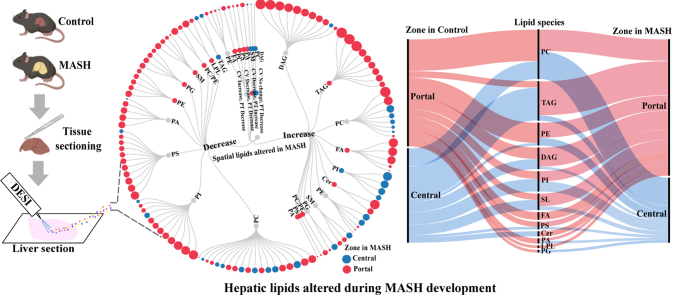

Metabolic dysfunction-associated fatty liver disease (MASLD) is the most prevalent chronic liver disease worldwide, with a substantial increase in the global prevalence, rising to 38%. It is closely linked to metabolic disorders, including obesity, type 2 diabetes mellitus, and dyslipidemia (1, 2). It is defined as an accumulation of fat in more than 5% of the hepatocytes without relevant alcohol consumption and in the absence of concomitant liver pathology (3). It can progress to metabolic dysfunction-associated steatohepatitis (MASH). The pathogenesis of MASLD involves a complex interplay of various metabolic and signaling pathways, progressing gradually over an extended period of time. The initial stage of MASLD pathogenesis consists of accumulating triacylglycerols (TAG) in hepatocytes, known as steatosis (4). As the intrahepatic production of TAG exceeds its secretion in the form of very low-density lipoproteins, lipid droplets accumulate in hepatocytes (5, 6). MASH is histologically defined by the presence of steatosis as well as additional features, including hepatocyte ballooning and lobular inflammation with or without perisinusoidal fibrosis (3, 7). The development of MASH is a complex process influenced by various concurrent factors. Genetic factors, dietary habits, and environmental and sedentary lifestyles can collectively contribute to developing insulin resistance, obesity, and alterations in the intestinal microbiome (8, 9). These factors, along with multiple hits such as oxidative stress, impaired mitochondrial lipid oxidation, endoplasmic reticulum stress, and inflammation, contribute to MASH development (10). A recent systematic review and meta-analysis reported that approximately 31% of patients diagnosed with MASLD progressed to MASH within a median duration of 4.7 years (11). In addition, undiagnosed MASH can silently progress to advanced liver disease without noticeable symptoms, eventually leading to advanced cirrhosis and hepatocellular carcinoma.

Our understanding of lipid metabolism in MASH has advanced due to an advancement in the field of lipidomics. Several studies investigated lipid metabolism in MASH in circulation (serum or plasma) and liver tissue homogenates (12, 13, 14, 15). These studies have provided evidence suggesting that dysregulation of lipid metabolism within the liver may play a pivotal role in the pathogenesis of MASH (16). Furthermore, other reports have provided evidence regarding the potential involvement of fatty acids (FA) (14, 15), TAGs (13, 17), phospholipids (17), and sphingolipids (13, 17, 18) in the progression of MASH. While several studies have proposed potential biomarkers for disease progression (19, 20), further research is required to understand the altered lipid biosynthesis, metabolism, and signaling in MASH.

The liver comprises numerous hexagonal structures known as hepatic lobules, which consist of hexagonal portal tracts surrounding a central vein. Within liver lobules, oxygen, and nutrient levels vary from the periportal to the pericentral area, resulting in hepatocytes with distinct phenotypes and metabolic functions and creating liver zonation (21, 22, 23). Consequently, conventional lipidomics analyses using liver homogenates do not provide sufficient information regarding hepatocytes’ phenotype across liver zonation. We have previously developed molecular mass spectrometry imaging to overcome this limitation and evaluate the zone-specific hepatic lipid metabolism distribution (21). Despite previous reports on the heterogeneous function of lipid metabolism in hepatocytes (24, 25, 26, 27), our understanding of zone-specific phenotypes within the liver during MASH is limited. Here, we hypothesized that altering zone-specific lipid metabolism could contribute to pathophysiological changes during MASH development. To test our hypothesis, we developed an already established Western diet (WD) combined with a carbon tetrachloride (CCl_4_)-induced MASH model, which resembles human MASH both in terms of pathology and altered metabolic pathways (28, 29). We explored zone-specific lipid metabolism across liver zonation in the MASH model using desorption electrospray ionization mass spectrometry imaging (DESI-MSI). In addition, we assessed the FA composition of altered lipid species, particularly in relation to zonation.

## Material and Methods

### Experimental model and liver collection

Male C57Bl6/J mice aged eight weeks (Charles River) were housed in groups of five per cage and maintained on a 12-h light-dark cycle with a controlled temperature of 22 ± 2°C. The mice were acclimated to the housing facility for one week before the commencement of the study. Ten mice were randomly assigned to either the standard diet (Catalogue number 5053, LabDiet) or the Western diet, which contained 42% Kcal/fat, including sucrose and 1.25% cholesterol (Catalogue number TD.120528, Envigo Teklad) for 18 weeks. They were randomly designated as the control (n = 5) or MASH (n = 5) group, respectively. The MASH group was also injected intraperitoneally with carbon tetrachloride (CCl_4_) (0.32 mg/kg) once per week, while the control group remained untreated throughout the study period. Food intake, body weight, and health status were recorded weekly. The mice were anesthetized with pentobarbital (100 mg/kg, i.p.) and euthanized in the afternoon to collect liver tissue. The liver tissue was snap-frozen and kept at −80°C. All experiments were performed following the regulations approved by the Bern Animal Welfare Committee (BE42/19).

### Liver tissue preparation

The frozen liver was sectioned at −21°C using the HYRAX C60 Cryostat machine (Zeiss, Germany) without embedding material. The tissue slices were thaw-mounted on glass slides (Thermo Scientific™) and stored at −80°C until the analysis. Tissue sections were obtained at 5 and 10 μm thicknesses, with the 5 μm sections used for histological analysis and the 10 μm sections utilized for DESI data acquisition.

### Histology analysis

The frozen liver tissue sections underwent standard staining procedures, including hematoxylin and eosin (H&E), Oil Red O, and Sirius red staining. For immunofluorescence, staining was performed as stated before (21). Briefly, the sections were fixed with 4% paraformaldehyde, washed with Bond™ wash solution, followed by antigen retrieval using EDTA/Tris and blocking with Opal™ blocking buffer. Immunostaining was performed using specific antibodies against glutamine synthetase (GS-6), which is selectively expressed in pericentral hepatocytes (1:10,000, Sigma-Aldrich, catalog G2781), E-cadherin (E-Cad) for periportal hepatocytes (1:200, Santa Cruz Biotechnology, Inc., catalog sc-7870), and DAPI to stain the nucleus of the hepatocyte. The slides were scanned using a Panoramic 250 Flash II slide scanner with a 20x objective (3DHISTECH Ltd) to generate optical images. An experienced liver pathologist evaluated the histological analysis and liver zonation classification. The histology results were quantified using MetaMorph software V7.8.12.0 (Molecular Devices, LLC).

### DESI-MSI data acquisition

Prior to DESI-MSI analysis, the liver slide was air-dried at room temperature for 15 min, and an optical image of the tissue was captured using a CanoScan LiDE 210 scanner (Canon, Tokyo, Japan). The slide was placed onto the two-dimensional moving stage holder. For positive ionization mode, the slide was gently immersed in 25 mM ammonium acetate for 20 s and air-dried for 45 min at room temperature before being placed on the stage.

A 2D Omni spray stage (Waters Corporation) coupled with Xevo G2-XS QTof (Waters Corporation, UK) was employed for spatial lipid imaging described previously (21). Briefly, the DESI-MSI imaging was acquired in positive and negative ionization mode over the mass range of m/z 100 to 1,200 at a spatial resolution of 50 μm and a scan rate of 200 μm/s. The sprayer incidence angle was set at 75°, with a distance of 2 mm between the sprayer and the tissue surface and 1 mm between the sprayer and the inlet. The charged spray solvent consisted of 98% methanol (Biosolve Chimie) with MilliQ water (Merck Millipore), which was sprayed at a flow rate of 2.0 μl/min. The capillary temperature was set at 150°C, and the capillary voltage was set at 0.6 kV in both positive and negative ionization modes. The sampling cone voltage was set at 120 V for positive and 110 V for negative ionization. The nebulizing gas (Nitrogen) was set to 8.5 PSI. The heated transfer line (HTL) inlet was positioned at an angle of 10° with a distance of 0.5 mm from the tissue surface, with HTL voltages set at 11 kV and 13 kV for positive and negative ionization modes, respectively.

MassLynx™ Software V4.2 (Waters Corporation) was used for data acquisition and spectrum preview. DESI ion images were visualized using the High Definition Imaging (HDI™) software V1.6 (Waters Corporation).

### DESI-MSI data analysis and lipids annotation

The DESI-MSI data were imported into LipostarMSI V1.1.0b28 (30) (Molecular Horizon srl, Italy) to perform data pre-processing. Briefly, pre-processing of the DESI-MSI data included peak alignment, profile smoothing, baseline correction, and peak picking (30). The intensity of the data set was normalized by total ion count (TIC). Tentative identification of lipids based on the accurate mass was accomplished by searching against LIPID MAPS databases (https://www.lipidmaps.org), and then lipid identification was confirmed by DESI-MS/MS analysis and Direct infusion tandem mass spectrometry (28).

Regions of interest (ROIs) were manually created by co-registration with histological data to determine spatial hepatic lipids using HDI software. The liver zonation in control and MASH samples were classified into zone 1, periportal zone, and zone 3, pericentral zone. Three different ROIs were selected in each liver zonation. Each region consisted of 10 pixels used to measure the amount of lipids in each ROI. Thus, for each zone, 30 pixels were considered to assess lipid intensity in each zone. For the analysis, the medians of lipid intensity in each of the three ROIs were considered. Univariate and multivariate statistical analyses were performed to identify lipids differentially expressed across liver zonation. A repeated-measure Analysis of Covariance (ANCOVA) was performed for each lipid with zonation (portal or central) and disease condition (control or MASH) as a fixed effect and mouse identifier as a random effect. The *P*-value of the alteration of the lipids was reported. Benjamini-Hochberg False Discovery Rate (FDR) was estimated to account for multiple tests. All the statistical analyses were analyzed using R version 4.0.2. The mean intensity and mean values of the fold change of the significant lipids detected in the periportal and pericentral zones of control and MASH samples were used to generate heat maps, boxplots, and Alluvial diagrams. The relative intensity percentage was calculated based on the highest intensity of the significantly altered lipid during MASH development, PI(18:0_20:4), located in the pericentral area of the control group. This percentage was then used to create two-sided bar plots.

## Results

### A Western diet with CCl_4_-induced MASH in the experimental model

**Fig. 1:**
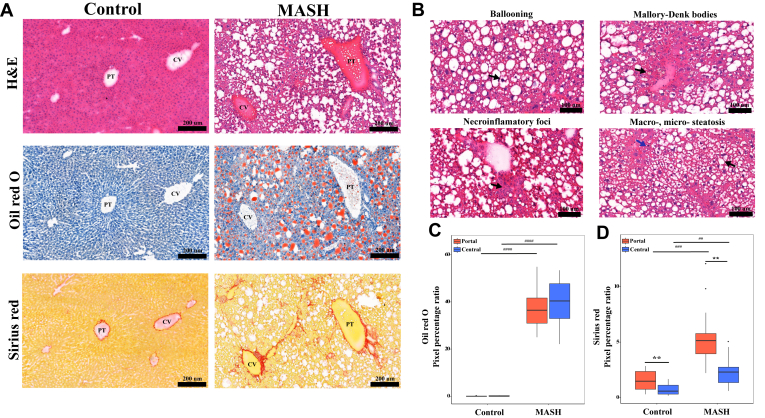
Histological staining showed WD diet- and chemical-induced MASH. A: the H & E, Oil Red O, and Sirius red staining of the portal (PT) and central (CV) areas of the control and MASH livers (20X). B: the H&E staining showed MASH pathophysiology, including ballooning, Mallory-Denk bodies, inflammation and accumulation of macro- (black arrow) and micro-vascular fat (blue arrow) in the MASH liver. C: The quantification of the oil red o staining in pixel percentage ratio. D: The quantification of the Sirius red staining in pixel percentage ratio. ^##^*P-value* < 0.01, ^###^*P-value* < 0.001 and ^####^*P-value* < 0.0001 the MASH compared with the control, ∗∗ *P-value* < 0.01 the pericentral areas of each group compared with the periportal areas.

It was previously shown that the diet- and chemical-induced MASH model resembles human MASH pathophysiology and hepatic lipid metabolism (28, 29). Thus, we used this animal model to investigate zone-specific hepatic lipid metabolism. The mice were randomly assigned to either the control group, fed a standard diet, or the MASH group, fed a WD diet for 18 weeks plus CCl_4_ once a week. Histological analysis using H&E staining revealed the MASH pathophysiology, including accumulation of macro- and micro-vascular fat, ballooning, Mallory-Denk bodies, and inflammation in the liver of the MASH group, as shown in Fig. 1A, B. The Oil Red O staining demonstrated a significant 40% increase in neutral lipids in both the periportal and pericentral areas of the MASH group, as illustrated in Fig. 1A, C. No significant difference was observed in the levels of neutral lipids between the periportal and pericentral areas. Fibrosis level, measured using Sirius red staining, was significantly increased in the MASH group compared to the control. We observed a 4-fold and 3-fold increase in the periportal and pericentral regions of MASH, respectively (Fig. 1A, D). The periportal areas exhibited a 2-fold increase in fibrosis than the pericentral areas in both control and MASH groups (Fig. 1D).

### Hepatic lipids alteration and localization in control versus MASH liver

Following our initial observation regarding alteration in lipid metabolism during MASH (28), we employed the developed and validated spatial metabolic imaging technique using DESI-MSI (21) to investigate the alterations and the spatial distribution of hepatic lipids across the zonation of the control and MASH livers. We identified 287 distinct lipid species (Supplemental Table S1) from control and MASH liver tissues, encompassing a diverse range of lipid classes such as FAs, lysophospholipids (LPLs), PAs, phosphatidylcholines (PCs), phosphatidylethanolamines (PEs), phosphatidylglycerols (PGs), phosphatidylinositols (PIs), phosphatidylserines (PSs), diacylglycerols (DAGs), TAGs, ceramides (Cers) and sphingolipids (SLs). A total of 130 out of 287 lipids were found to be significantly altered in the MASH model (Supplemental Table S2).

**Fig. 2:**
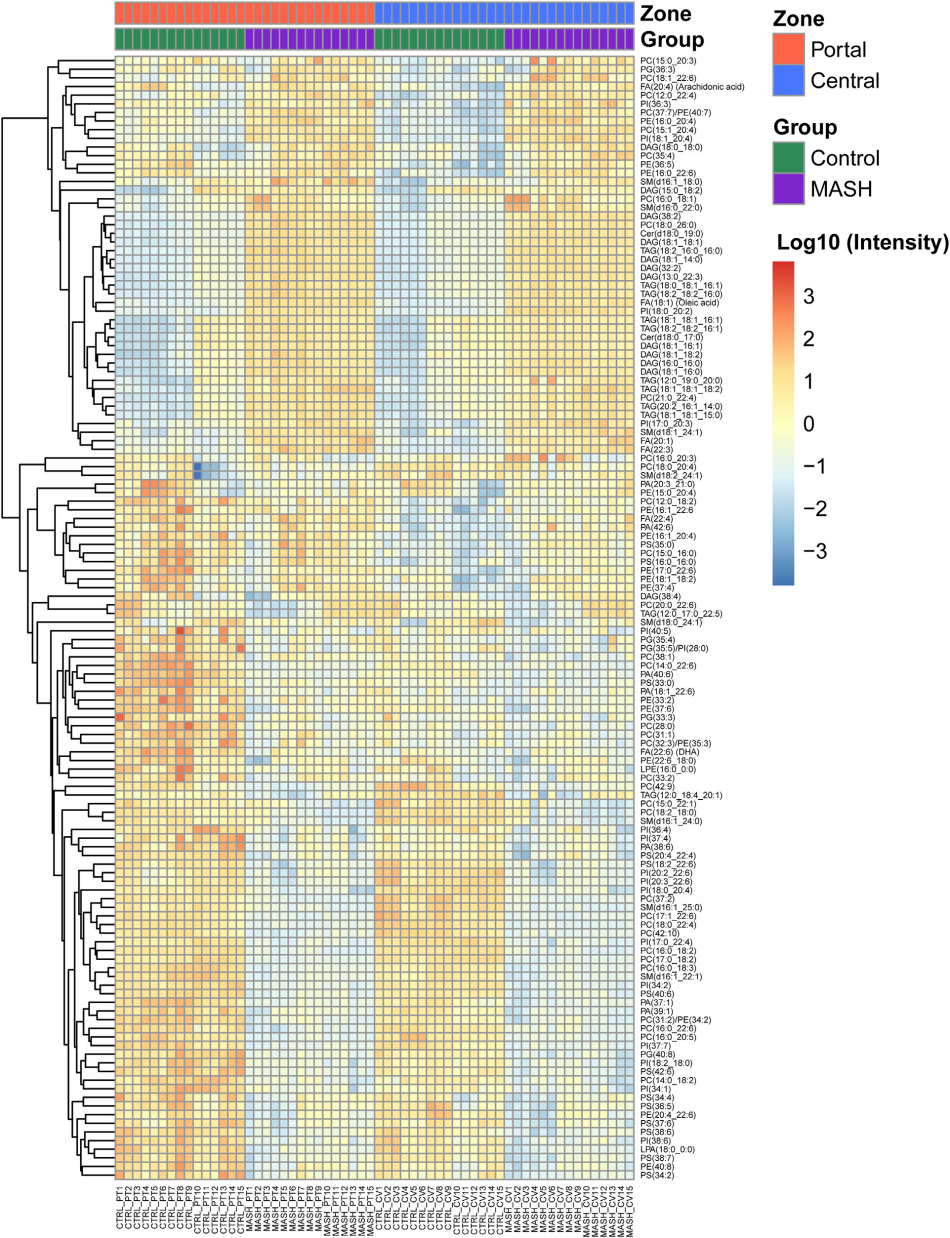
Heatmap illustrating significant alterations of hepatic lipids across periportal (PT) and pericentral (CV) zones in control and MASH samples.

**Fig. 3:**
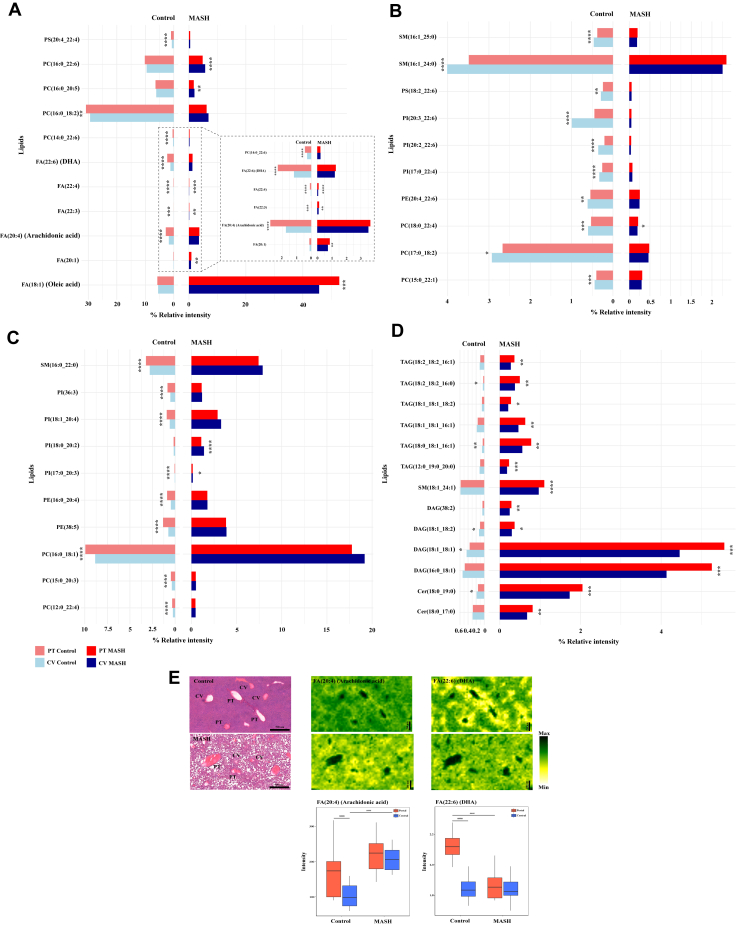
Zone-specific localisation of the significant hepatic lipids altered in control and MASH. A–D: Two-sided bar plots illustrate lipid alterations across two zones (periportal, PT, and pericentral, CV) in control and MASH samples. A and B: Zone-specific localization of the decreasing lipids in the MASH stage. C and D: Zone-specific localization of the increasing lipids in the MASH stage. E: Ion images and box plots displayed the spatial distribution of FA20:4 and FA 22:6 in the periportal (PT) and pericentral (CV) of control and MASH samples. ∗*P-value* < 0.05, ∗∗*P-value* < 0.01, ∗∗∗*P-value* < 0.001 and ∗∗∗∗*P-value* < 0.0001 the pericentral areas of each group compared with the periportal areas, ^####^*P-value* < 0.0001 the MASH compared with the control (box plot (E)).

Figure 2 illustrates the spatial changes in hepatic lipid distribution across the periportal and pericentral zones in both control and MASH groups. We observed significant changes in various classes of lipids, including FAs, PAs, PCs, PEs, PGs, PIs, PSs, DAGs, TAGs, Cers and SLs across the periportal and pericentral zones in both control and MASH liver. The two-sided bar plots (Fig. 3A–D) visually represent significant lipid alterations in both control (indicated on the left side of the bar plots) and MASH (displayed on the right side of the bar plots) while also illustrating the spatial distribution of these lipids across the periportal (highlighted in red) and pericentral (highlighted in blue) zones. Some lipid species, including PC(14:0_22:6), PC(15:0_22:1), PC(16:0_18:2), PC(16:0_20:5), PC(16:0_22:6), PC(17:0_18:2), PC(18:0_22:4), PE(20:4_22:6), PI(17:0_22:4), PI(20:2_22:6), PI(20:3_22:6), PS(18:2_22:6), PS(20:4_22:4), SM(16:1_24:0) and SM(16:1_25:0) decreased significantly in both periportal and pericentral regions in the MASH group compared to the control (Fig. 3A, B). Notably, PC(16:0_20:5) and PC(16:0_22:6) were distributed homogenously across the liver zonation in the control group with no significant differences between zones (Fig. 3A). However, we observed statistically significant differences in these lipids across liver zonation in the MASH group. They showed higher levels in the pericentral zone than the periportal zone of the MASH group (Fig. 3A). Conversely, PC(14:0_22:6), PC(15:0_22:1), PC(16:0_18:2), PC(17:0_18:2), PE(20:4_22:6), PI(17:0_22:4), PI(20:2_22:6), PI(20:3_22:6), PS(18:2_22:6), PS(20:4_22:4), SM(16:1_24:0) and SM(16:1_25:0) were distributed significantly differently across liver zonation in control but not the MASH group (Fig. 3A, B). PC(14:0_22:6), PC(16:0_18:2) and PS(20:4_22:4) were detected predominantly in the periportal zone, while PC(15:0_22:1), PC(17:0_18:2), PE(20:4_22:6), PI(17:0_22:4), PI(20:2_22:6), PI(20:3_22:6), PS(18:2_22:6), SM(16:1_24:0) and SM(16:1_25:0) were higher in the pericentral zone of the control group (Fig. 3A, B). Additionally, PC(18:0_22:4), predominantly located in the pericentral zone in the control group, was detected mainly in the periportal zone of the MASH group (Fig. 3B).

On the other hand, several lipid species increased significantly in MASH and exhibited differential distribution across liver zonation, as shown in Fig. 3C, D. More specifically, Cer(18:0_17:0), DAG(16:0_18:1), DAG(38:2), PI(18:0_20:2), SM(18:1_24:1), TAG(12:0_19:0_20:0), TAG(18:1_18:1_16:1), TAG(18:1_18:1_18:2), and TAG(18:2_18:2_16:1) exhibited distinct distribution across the liver zonation in MASH liver, while this was not evident in the control group (Fig. 3C, D). The PI(18:0_20:2) were found at a higher level in the pericentral zone of the MASH group (Fig. 3C). Cer(18:0_17:0), SM(18:1_24:1), DAG(16:0_18:1), DAG(38:2), TAG(12:0_19:0_20:0), TAG(18:1_18:1_16:1), TAG(18:1_18:1_18:2) and TAG(18:2_18:2_16:1) were mainly located in the periportal zone of the MASH group (Fig. 3D). Conversely, SM(16:0_22:0), PC(12:0_22:4), PC(15:0_20:3), PC(16:0_18:1), PE(38:5), PE(16:0_20:4), PI(18:1_20:4) and PI(36:3) displayed distinct alterations in the control group but not in the MASH group (Fig. 3C). They were predominantly expressed in the periportal zone of the control. Moreover, Cer(18:0_19:0), DAG(18:1_18:1), DAG(18:1_18:2), TAG(18:0_18:1_16:1), TAG(18:2_18:2_16:0) and PI(17:0_20:3) displayed heterogeneous distribution across the liver zonation in both the control and MASH samples (Fig. 3C, D). The PI(17:0_20:3) was detected primarily in the control group's periportal zone and the MASH group's pericentral zone (Fig. 3C). Cer(18:0_19:0), DAG(18:1_18:1), DAG(18:1_18:2), TAG(18:0_18:1_16:1) and TAG(18:2_18:2_16:0) were mainly located in the pericentral zone of the control group, while predominantly expressed in the periportal zone of the MASH group (Fig. 3D).

As illustrated in Fig. 3A, significant changes were observed in FFAs (free fatty acid) across liver zonation of the control and MASH groups. In the MASH group, we observed a significant increase in FA(18:1), FA(20:1), and FA(22:3) levels in the periportal and pericentral regions compared to the control group. Specifically, distinct elevation in the level of FA(20:4) (Fig. 3A, E) and reduction in FA(22:4) was exclusively observed in the pericentral zone. At the same time, FA(22:6) (Fig. 3A, E) decreased exclusively in the periportal zone in the MASH group. FA(18:1) and FA(20:1) also exhibited unequal localization across the liver zones in the MASH, which was predominantly expressed in the periportal zones (Fig. 3A). In contrast, FA(20:4) and FA(22:6) showed predominantly heterogeneous localization in the control group in the periportal zones. Conversely, FA(22:3) and FA(22:4) consistently observed uneven distribution in control and MASH samples. They were mainly located in the periportal zones of both the control and MASH groups.

### Zone-specific hepatic lipids alteration and remodeling in MASH development

**Fig. 4:**
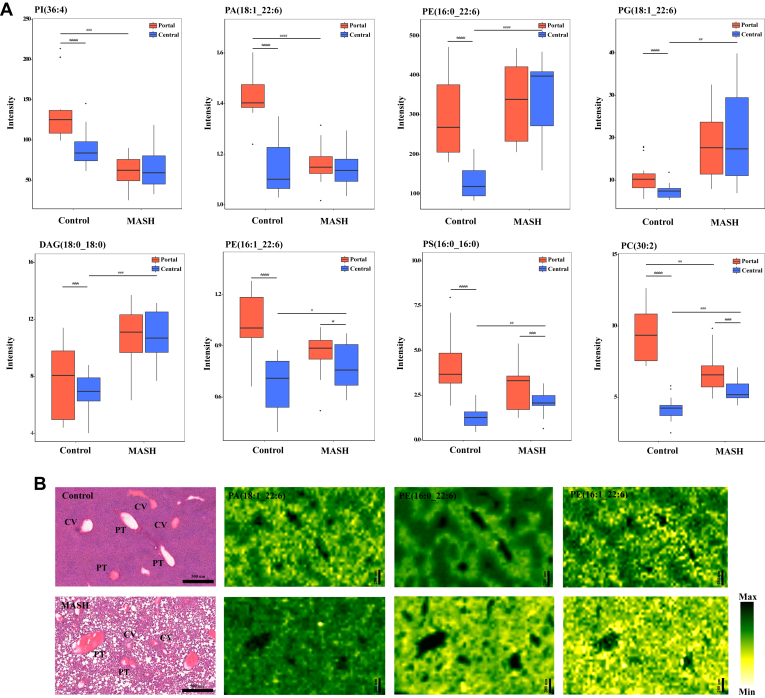
Zone-specific perturbations of hepatic lipids alteration in control and MASH. A: Box plots illustrating the zone-specific alteration of lipids across the liver zonation in control and MASH livers, including PI(36:4), PA(18:1_22:6), PE(16:0_22:6), PG(18:1_22:6), DAG(18:0_18:0), PE(16:1_22:6), PS(16:0_16:0) and PC(30:2). B: Ion images displaying the spatial distribution of PA(18:1_22:6), PE(16:0_22:6), and PC(30:2) in control and MASH samples. In this figure, the H&E staining images of the control and MASH groups are derived from the same H&E staining of the same samples as Fig. 3E. These images are reused across figures due to their representation of consistent staining results for comparative analysis. ^#^*P-value* < 0.05, ^##^*P-value* < 0.01, ^###^*P-value* < 0.001 and ^####^*P-value* < 0.0001 the MASH compared with the control, ∗*P-value* < 0.05, ∗∗∗*P-value* < 0.001 and ∗∗∗∗*P-value* < 0.0001 the pericentral areas of each group compared with the periportal areas.

We further investigated the integrity of metabolic zonation in MASH. As demonstrated in Fig. 4A, zone-specific patterns of some lipids were perturbed during MASH. Specifically, PI(36:4) and PA(18:1_22:6) lost the zone-specific distribution in MASH, which was observed in the control liver. This resulted from an exclusive reduction in the periportal region compared to the control, while the levels remained unchanged in the pericentral region, leading to equal distribution across liver zonation. On the other hand, PE(16:0_22:6), PG(18:1_22:6), DAG(18:0_18:0), PE(16:1_22:6) and PS(16:0_16:0) exhibited zone-specific distribution in the control group. Among these lipids, only PE(16:1_22:6) and PS(16:0_16:0) displayed heterogeneous distribution in both control and MASH samples. Furthermore, the levels of PE(16:0_22:6), PG(18:1_22:6), DAG(18:0_18:0), PE(16:1_22:6) and PS(16:0_16:0) were significantly increased exclusively in the pericentral zone, with no significant difference observed in the periportal zone. Interestingly, PC(30:2) showed distinct distribution in both control and MASH samples. However, it displayed opposite alterations in the two zones. In MASH, PC(30:2) decreased in the periportal area while increasing in the pericentral region compared to the control. Figure 4B demonstrates the examples of ion images of these lipid species.

**Fig. 5:**
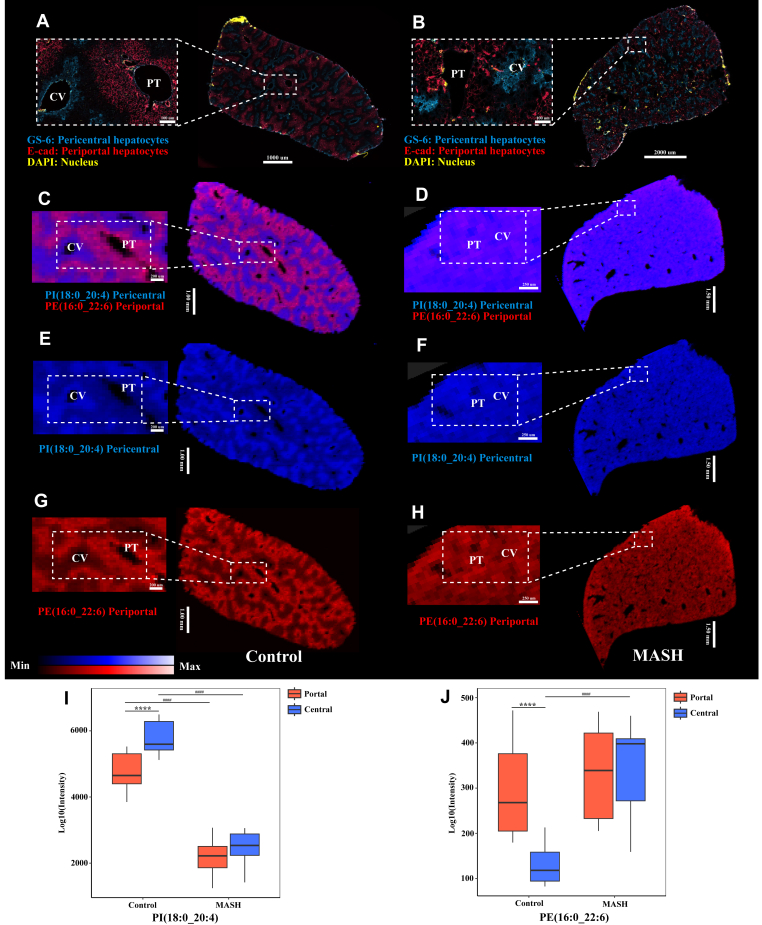
The zonation patterns observed in control and MASH livers by double-immunofluorescence staining and molecular imaging with DESI. Double-immunofluorescence staining shows the proto-central axis and the liver zonation of control (A) and MASH (B) livers. GS-6, pericentral hepatocytes (blue); E-Cad, periportal hepatocytes (red); and DAPI (yellow). Red and blue overlay ion images from DESI-MSI: PI(18:0_20:4) located in the pericentral area (blue) and PE(16:0_22:6) predominantly presented in the periportal region (red) of control (C) and MASH (D) livers. Ion images displayed spatial distribution obtained from control and MASH mice show the predominantly localisation of PI(18:0_20:4) in the pericentral (E, control) and (F, MASH) and PE(16:0_22:6) in the periportal (G, control) and (H, MASH). CV, central vein; PT, Portal tried; GS-6, glutamine synthetase; E-Cad, E-cadherin. Box plots visualising the zone-specific alteration of PI(18:0_20:4) (I) and PE(16:0_22:6) (J) across the liver zonation in control and MASH livers. The box plot data for PE(16:0_22:6) presented in the Figure are the same as those in Fig. 4A. This reuse is intended to highlight the lipid's specific localisation and changes in the MASH group's pericentral areas. ####*P*-value < 0.0001 the MASH compared with the control, ∗∗∗∗*P*-value < 0.0001 the pericentral areas of each group compared with the periportal areas.

We further examined the zonation pattern of the liver in control and MASH samples using double immunofluorescence staining for pericentral and periportal hepatocytes, as demonstrated in Fig. 5A, B. Consistent with the immunofluorescence staining results, a distinct zonation pattern of specific lipids across the liver lobule in the control liver was observed, as illustrated by ion images from DESI-MSI (Fig. 5C). In contrast, the zonation pattern was not clearly observed in the MASH liver (Fig. 5D). This possibly indicates an alteration in zone-specific lipid distribution during the MASH stage. For example, in the control liver (Fig. 5E, I), PI(18:0_20:4) was primarily localized in the pericentral area. However, during MASH (Fig. 5F, I), the level of PI(18:0_20:4) dramatically decreased in the pericentral zones, leading to its equal distribution across the MASH liver. Additionally, an increase in PE(16:0_22:6) levels, specifically in the pericentral area, was detected in the MASH livers, resulting in its equal distribution across the MASH liver, as demonstrated in Fig. 5G, H, J.

### Changes in the specific fatty acid composition associated with hepatic lipid alterations in MASH

**Fig. 6:**
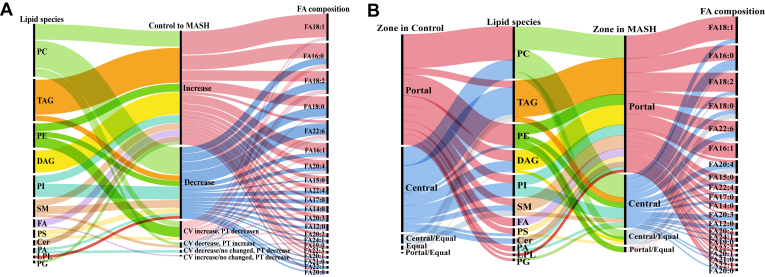
Alluvial diagram depicting FA chain composition in the significant hepatic lipids altered and exhibited spatial changes predominantly during MASH. A: The FA compositions of hepatic lipids with altered levels (increased and decreased) in the MASH group compared to the control. B: The FA compositions of hepatic lipids exhibited significant spatial changes predominantly within specific zones in the MASH group compared to the control.

We further investigated the composition of fatty acids in the significantly altered hepatic lipids in the MASH group (Fig. 6). The TAG, DAG, FA, and Cer levels dramatically increased, while the phospholipids decreased in the MASH liver compared to the control. Most increased lipid species in the MASH liver contained saturated (SFA) and monounsaturated (MUFA) FA, including FA(16:0), FA(18:0), FA(16:1) and FA(18:1) (Fig. 6A). Conversely, polyunsaturated FAs such as FA(22:6) and FA(22:4) were the major FA composition of the reduced hepatic lipids in MASH (Fig. 6A). However, arachidonic acid (FA(20:4)) was one of the predominant FA composition in the elevated lipids in the MASH group.

The Alluvial diagrams (Fig. 6B) illustrated the FA composition of the alteration of the significant lipids across liver zonation (the portal and the central zones) in the MASH compared to the control groups. Our observations highlight the significant involvement of SFA and MUFA, including FA(16:0), FA(16:1), FA(18:0), FA(18:1), and FA(18:2), as key constituents of the FA composition within the hepatic lipids that predominantly change their spatial distribution to the periportal region in MASH.

Notably, as demonstrated in Fig. 6B, most of TAG, DAG, SM, and Cer, such as TAG(18:2_18:2_16:0), DAG(18:1_18:1), SM(18:1_24:1), and Cer(18:0_19:0), transitioned the predominant location from the pericentral region in control to the periportal region in MASH. Conversely, phospholipids such as PC(18:0_22:4) and PI(17:0_20:3) were predominantly located in the periportal region in control. However, they were mainly detected in the pericentral area in the MASH stage.

## Discussion

Lipid metabolism plays a pivotal role in the development and progression of MASH (6, 16). Various preclinical mouse models of MASH, encompassing diet-induced, chemical-induced, and genetically modified variants, have emerged in the past decade, providing a platform to unravel the complex mechanism associated with the development of MASH (31, 32). Tsuchida *et al.* have previously demonstrated that diet- and chemical-induced MASH models resemble human MASH histological, immunological, and transcriptomic features (29). In addition, our previous study used transcriptomics-driven metabolic pathway analysis to demonstrate the similarity between the diet- and chemical-induced MASH model and human MASH, particularly concerning lipid metabolism (28). Thus, for this study, we use this MASH model to investigate zone-specific changes in lipid metabolism. We observed a significant increase in lipid accumulation (steatosis) by 40-fold, fibrosis by 4-fold, and ballooned hepatocytes in this established MASH model. According to Halpern *et al.*, around 50% of the genes expressed in the liver exhibit a non-random zone-specific distribution (25). Furthermore, our previous work has revealed the heterogeneity of lipid metabolism across liver zonation in healthy liver (21). In the present study, we investigated hepatic lipid alteration and zone-specific hepatic lipid metabolism in the MASH mouse model. We further assessed the alteration in non-esterified FAs and esterified FAs within several classes of lipids in the MASH model.

Using spatial lipidomics, we identified 130 lipids altered significantly in lipid abundance and localization across liver zonation in the MASH group compared to the control. Interestingly, several lipid species displayed zone-specific alterations in control and MASH, as shown in Fig. 2. We observed alteration in various lipid classes, including FAs, PAs, PCs, PEs, PGs, PIs, PSs, DAGs, TAGs, Cers, and SLs in MASH. Consistent with recent studies highlighting the zonation pattern of a healthy liver (21, 33, 34, 35), we observed a distinct zonation pattern for specific lipids across the liver lobule (Figs. 3 and 4). Furthermore, we observed perturbation in lipid zonation in MASH compared to the healthy liver. As mentioned, this is potentially related to altering and redistributing several lipids across different liver zones (Fig. 5). In addition, we detected an elevation in fibrosis in the periportal region of MASH compared to the pericentral, which could potentially contribute to the dissimilar zonation pattern in the MASH (36, 37).

In accordance with the present results, previous studies from Wattacheril *et al.* have demonstrated a significant reduction and distribution pattern change in phospholipids (PLs), especially PCs, during the MASLD progression (34). However, this study did not mention the FA composition of the altered lipids. Our findings reveal the zone-specific localization of PLs, including their FA composition, particularly in the control group. This zonation was partially or completely lost in the MASH group, which exhibited a substantial depletion of most PLs. Phospholipids are primary constituents of cellular membranes, and disruptions in their structural integrity can lead hepatic cellular membranes to be susceptible to various insults, such as hepatic lipotoxicity and immunological response and activators (38, 39). This increased susceptibility in the periportal and pericentral areas may contribute to inflammation and cell apoptosis during MASH development. Moreover, we identified the unique distribution of zone-specific perturbations during MASH in PI(36:4), PA(18:1_22:6), PE(16:0_22:6), PG(18:1_22:6), DAG(18:0_18:0), PE(16:1_22:6), PS(16:0_16:0) and PC(30:2).

Previous studies in both humans and rodents (40, 41, 42, 43) have demonstrated that excessive accumulation of TAG in the liver is primarily associated with the elevated transport of FA to the liver, as well as additional production of new lipids through de novo lipogenesis (DNL) (44). Our observation of heightened levels of free fatty acids (FFAs) in the periportal zone across both control and MASH samples (Figs. 3A and 6B) aligns with prior studies indicating increased fatty acid uptake and biosynthesis in periportal hepatocytes (24, 26, 27). This correlation hints at a potential association with elevated FFA concentrations in the bloodstream within this zone (45), likely influenced by blood flow from the periportal area to the pericentral area. Consequently, it establishes a gradient of FFA from the periportal to the porto-central region of the liver lobule. Additionally, using spatial lipidomics analysis, we observed the elevation levels of FFA and TAG, particularly in the periportal region of MASH samples (Fig. 3A, D), which could be attributed to the findings of Berndt *et al.* (24), who demonstrated that with higher plasma concentrations, as indicated in a source from WD (46), the uptake rate of pericentral hepatocytes reaches saturation, whereas the uptake capacity of periportal hepatocytes remains unaffected (24). Furthermore, the study highlighted that periportal hepatocytes exhibit greater FA and TAG synthesis capacity than pericentral hepatocytes, providing a plausible explanation for our observed lipid accumulation patterns (24).

Although the zonation in the MASH samples became less pronounced, through spatial lipidomics analysis, we observed a heterogeneous distribution of DAG and TAG (Figs. 3D and 6B), primarily located in the pericentral zones in the control group. However, during MASH development, their localization shifted predominantly to the periportal zones. This supports the histological evidence of steatosis, which results from excess accumulation of TAG and is typically more distributed in a distinctly pericentral area or zone 3 in the early stage of the disease (47). Our histological results from Oil Red O staining showed a significant increase in neutral lipids like TAGs in the MASH group but no significant difference in their distribution between the two zones of the control and MASH samples. Our data revealed predominant SFA compositions, specifically FA(12:0), FA(14:0), FA(15:0), FA(16:0), FA(18:0), FA(19:0) and FA(20:0), in the periportal zone of the MASH samples. Additionally, we also observed predominant fibrosis in the periportal areas of the MASH, which we proposed may be related to the lipotoxicity that produces and releases reactive oxygen species (48), leading to the activation of hepatic stellate cells and generating fibrosis in this area (49). Additional studies are required to confirm the mechanism that leads to elevated fibrosis in this zone.

Several spatial metabolic imaging studies, including SIMS and MALDI, demonstrated a specific distribution of hepatic lipids across liver zonation, mainly emphasizing TAG and phosphatidylcholines (33, 34, 35, 50), lacking comprehensive details on overall lipid metabolism and FA composition. Understanding the progression of MASLD in relation to insulin resistance, FA metabolism, and DNL also relates significantly to the FA composition in each lipid species. Indeed, the FA composition of phospholipids has only been documented in a study distinguishing lipid signatures between steatotic and non-steatotic areas (50). The current study demonstrated the FA composition of hepatic lipids and revealed significant alterations in the proportion of SFA, MUFA, and PUFA during MASH development. Most increased lipid species in MASH livers consist of SFA and MUFA composition, while the reduced lipid species in MASH contained mainly PUFA composition. Arachidonic acid was an exception, which increased exclusively in the MASH group. Similarly, the MUFA of FFAs such as FA(18:1) and FA(20:1) increased dramatically in the periportal and pericentral zones of the MASH group. In contrast, FFA(22:6), which was detected predominantly in the periportal zone in the control group, was decreased in MASH. Moreover, our findings demonstrate the FA composition of hepatic lipids that contain SFA and MUFA, including 16:0 and 18:0, 16:1, 18:1, and 18:2, which were predominantly localized in the pericentral region in control altered to the periportal area of MASH. Furthermore, the transformation of the predominant zonation from pericentral to periportal, especially in TAG, DAG, SL, and Cer, was observed in the MASH group. Conversely, the transition of several phospholipids primarily located in the periportal region in the control to the pericentral area in the MASH was observed.

Our findings demonstrated the shift in FA composition of hepatic lipids containing SFA and MUFA, such as 16:0, 18:0, 16:1, 18:1, and 18:2, towards the periportal area in MASH samples, reflecting the localization of key DNL enzymes. This is in agreement with previous observation by Evan *et al.* that DNL enzymes, including ATP citrate lyase (ACL), acetyl-CoA carboxylase (ACC), and fatty acid synthase (FASN), are predominantly expressed in the periportal zone (51). This is also in agreement with our previous observation, which indicated the changes in hepatic gene expression related to lipid metabolism, including fatty acids (uptake, biosynthesis, activation, desaturation, oxidation, and metabolism), DNL, phospholipids (biosynthesis and metabolism), and TAG biosynthesis in MASH (28). However, the zonation pattern of DNL within the liver lobule remains contradictory, with numerous studies consistently identifying its predominant occurrence in pericentral hepatocytes (45). In their follow-up investigation, Evan *et al.* (52) suggested that zonation in the fed state may not be the result of differences in DNL enzyme mass but rather differences in enzyme-specific activity, proposing a phosphorylation-mediated regulation of ACC activity, a rate-limiting enzyme in DNL (51, 52).

Acknowledging the limitations, it is important to note that the control group did not receive a vehicle injection or any additional handling, unlike the MASH group. While WD and CCl_4_ induce MASH in mice, CCl_4_-induced liver injury in mouse models can induce hepatocyte necrosis and inflammation, contributing to the development of MASH-like pathology. It is crucial to recognize that this approach may not fully replicate the multifactorial etiology of human MASH. In fact, this is a challenge with any animal model (31). The human MASH arises from a complex interplay of genetic factors, metabolic factors, and environmental influences, which may not be fully recapitulated by only diet and hepatotoxicity treatment alone. Despite these limitations, the results obtained from our MASH mouse model provide valuable insights into the spatial distribution of lipid metabolism and the underlying pathogenesis of MASH. By elucidating the changes in lipid metabolism associated with MASH-like pathophysiology, these findings contribute to our understanding of the disease mechanisms. Future investigations should incorporate several MASH mouse models that more closely mimic the complexity of human MASH. Additionally, considering the translational relevance of preclinical findings to ensure the development of effective therapeutic strategies for human MASH could be crucial.

In summary, our study assessed lipid metabolism across liver zonation and provided insights into FA composition in various lipids contributing to the development of MASH. Our results suggest that the alteration and perturbation of the spatial distribution of lipid metabolism could contribute to the development of MASH. Hepatic FFAs are predominantly taken up from circulation and adipose tissue in the periportal zones. Lipid droplets were primarily located in the pericentral (zone 3) regions (53, 54, 55). Multiple cellular stresses in lipid-loaded hepatocytes, including oxidative stress, inflammation, and apoptosis, may contribute to MASH development.

Spatial lipidomics analysis allowed us to investigate lipid liver zonation associated with MASH pathophysiology, particularly highlighting the lipids composed of SAFs and MUFAs in the periportal zones of the liver lobular associated with elevated fibrosis, DNL, and lipotoxicity. Understanding the zone-specific lipid metabolism could lead to a better understanding of the pathogenesis of MASH and provide better diagnostic markers potentially related to the zone-specific localization in the liver. The altered spatial lipid distribution observed during the MASH stage may have implications for the pathogenesis and progression of the disease. Further exploration of the mechanisms underlying these lipid redistribution patterns could provide valuable insights into the pathophysiology of MASH.

## Data Availability

The authors confirm that the data supporting the findings of this study are available within the article and supplementary material. Due to the confidentiality of data, other than those is only available on request to the corresponding author (Mo. M).

## Supplemental data

This article contains supplemental data.

## Conflict of interest

The authors declare that they have no conflicts of interest with the contents of this article.
