# Supplementary material for: Spatial lipidomics reveals zone-specific hepatic lipid alteration and remodeling in metabolic dysfunction-associated steatohepatitis
Source: J Lipid Res. 2024 Jul 18;65(9):100599. doi: 10.1016/j.jlr.2024.100599 (PMC11388789; doi:10.1016/j.jlr.2024.100599)
Supplement: Supplemental Data [file mmc1.docx]

**Supplementary Tables**

**Table S1:** List of identified spatial hepatic lipids in control and MASH samples using DESI-MSI

| **Lipids** | **Adduct** | **m/z** | **Lipid Species** |
| --- | --- | --- | --- |
| Cer(35:0) | [M+Na]+ | 576.54 | Cer |
| Cer(37:0) | [M+Na]+ | 604.55 | Cer |
| DAG(32:0) | [M+H-H2O]+ | 551.51 | DAG |
| DAG(32:1) | [M+H-H2O]+ | 549.49 | DAG |
| DAG(32:2) | [M+H-H2O]+ | 547.47 | DAG |
| DAG(33:2) | [M+Na]+ | 601.52 | DAG |
| DAG(34:1) | [M+H-H2O]+ | 577.54 | DAG |
| DAG(34:2) | [M+H-H2O]+ | 575.51 | DAG |
| DAG(34:3) | [M+H-H2O]+ | 573.48 | DAG |
| DAG(34:3) | [M+H]+ | 591.50 | DAG |
| DAG(35:2) | [M+H-H2O]+ | 589.52 | DAG |
| DAG(35:3) | [M+H]+ | 605.52 | DAG |
| DAG(35:5) | [M+NH4]+ | 618.53 | DAG |
| DAG(35:5) | [M+Na]+ | 623.51 | DAG |
| DAG(35:6) | [M+H]+ | 599.55 | DAG |
| DAG(36:0) | [M+H]+ | 625.56 | DAG |
| DAG(36:2) | [M+H-H2O]+ | 603.54 | DAG |
| DAG(36:3) | [M+H]+ | 619.55 | DAG |
| DAG(36:4) | [M+H]+ | 617.51 | DAG |
| DAG(36:5) | [M+H]+ | 615.50 | DAG |
| DAG(38:2) | [M+H-H2O]+ | 631.57 | DAG |
| DAG(38:4) | [M+H-H2O]+ | 627.54 | DAG |
| DAG(38:5) | [M+H]+ | 643.53 | DAG |
| FA 16:1 (Palmitoleic acid) | [M-H]- | 253.22 | FA |
| FA 18:1 (Oleic acid) | [M-H]- | 281.25 | FA |
| FA 18:2 (Linoleic acid) | [M-H]- | 279.23 | FA |
| FA 20:1 | [M-H]- | 309.28 | FA |
| FA 20:2 | [M-H]- | 307.26 | FA |
| FA 20:3 | [M-H]- | 305.25 | FA |
| FA 20:4 (Arachidonic acid) | [M-H]- | 303.23 | FA |
| FA 22:3 | [M-H]- | 333.28 | FA |
| FA 22:4 | [M-H]- | 331.26 | FA |
| FA 22:6 (DHA) | [M-H]- | 327.23 | FA |
| FA(19:0) | [M-H]- | 297.24 | FA |
| FA(19:1) | [M-H]- | 295.23 | FA |
| FA(19:2) | [M-H]- | 293.21 | FA |
| LPA(18:0) | [M-H]- | 437.26 | LPL |
| LPC(16:0) | [M+H]+ | 496.34 | LPL |
| LPC(18:0) | [M+H]+ | 524.37 | LPL |
| LPC(18:1) | [M+H]+ | 522.36 | LPL |
| LPC(18:2) | [M+H]+ | 520.34 | LPL |
| LPC(18:3) | [M+H]+ | 518.32 | LPL |
| LPE(16:0) | [M-H]- | 452.27 | LPL |
| LPE(18:0) | [M-H]- | 480.30 | LPL |
| **Lipids** | **Adduct** | **m/z** | **Lipid Species** |
| LPE(18:1) | [M-H]- | 478.29 | LPL |
| LPI(18:0) | [M-H]- | 599.31 | LPL |
| PA(34:1) | [M-H]- | 673.49 | PA |
| PA(36:1) | [M-H]- | 701.51 | PA |
| PA(37:1) | [M-H]- | 715.51 | PA |
| PA(38:4) | [M-H]- | 723.48 | PA |
| PA(38:6) | [M-H]- | 719.45 | PA |
| PA(39:1) | [M-H]- | 743.55 | PA |
| PA(40:3) | [M-H]- | 753.54 | PA |
| PA(40:4) | [M-H]- | 751.52 | PA |
| PA(40:6) | [M-H]- | 747.50 | PA |
| PA(40:7) | [M-H]- | 745.48 | PA |
| PA(41:3) | [M-H]- | 767.54 | PA |
| PA(42:6) | [M-H]- | 775.51 | PA |
| PC(20:3) | [M+H]+ | 546.36 | PC |
| PC(20:4) | [M+H]+ | 544.34 | PC |
| PC(26:0) | [M+H]+ | 650.45 | PC |
| PC(28:0) | [M+Cl]- | 712.47 | PC |
| PC(30:1) | [M-H]- | 688.53 | PC |
| PC(30:2) | [M+Cl]- | 736.47 | PC |
| PC(31:0) | [M-H]- | 718.53 | PC |
| PC(31:1) | [M-H]- | 752.50 | PC |
| PC(31:2)/PE(34:2) | [M-H]- | 714.49 | PC/PE |
| PC(32:0) | [M+H]+ | 734.59 | PC |
| PC(32:1) | [M+H]+ | 732.57 | PC |
| PC(32:2) | [M-H]- | 714.53 | PC |
| PC(32:3)/PE(35:3) | [M-H]- | 726.49 | PC/PE |
| PC(33:1) | [M+H]+ | 746.60 | PC |
| PC(33:1) | [M+Cl]- | 780.52 | PC |
| PC(33:2) | [M+Cl]- | 778.50 | PC |
| PC(34:0) | [M+H]+ | 762.62 | PC |
| PC(34:1) | [M+H]+ | 760.58 | PC |
| PC(34:1) | [M+Cl]- | 794.54 | PC |
| PC(34:2) | [M+H]+ | 758.56 | PC |
| PC(34:3) | [M+H]+ | 756.58 | PC |
| PC(34:4) | [M+H]+ | 754.56 | PC |
| PC(35:0) | [M+H]+ | 776.60 | PC |
| PC(35:1) | [M+H]+ | 774.59 | PC |
| PC(35:2) | [M+H]+ | 772.58 | PC |
| PC(35:3) | [M+H]+ | 770.53 | PC |
| PC(35:3) | [M+Cl]- | 804.52 | PC |
| PC(35:4) | [M+H]+ | 768.58 | PC |
| PC(35:5) | [M-H]- | 764.51 | PC |
| PC(36:0) | [M+H]+ | 790.60 | PC |
| PC(36:1) | [M+H]+ | 788.57 | PC |
| PC(36:1) | [M+K]+ | 826.57 | PC |
| PC(36:2) | [M+H]+ | 786.60 | PC |
| **Lipids** | **Adduct** | **m/z** | **Lipid Species** |
| PC(36:2) | [M+Cl]- | 820.55 | PC |
| PC(36:3) | [M+H]+ | 784.58 | PC |
| PC(36:4) | [M+H]+ | 782.57 | PC |
| PC(36:4) | [M+Cl]- | 816.52 | PC |
| PC(36:5) | [M+H]+ | 780.55 | PC |
| PC(36:5) | [M+Cl]- | 814.50 | PC |
| PC(36:6) | [M+H]+ | 778.56 | PC |
| PC(36:6) | [M+Cl]- | 812.49 | PC |
| PC(37:1) | [M+H]+ | 802.60 | PC |
| PC(37:2) | [M+H]+ | 800.61 | PC |
| PC(37:3) | [M+H]+ | 798.56 | PC |
| PC(37:4) | [M-H]- | 780.58 | PC |
| PC(37:4) | [M+H]+ | 796.53 | PC |
| PC(37:5) | [M+H]+ | 794.56 | PC |
| PC(37:6) | [M+H]+ | 792.59 | PC |
| PC(38:1) | [M-H]- | 798.65 | PC |
| PC(38:2) | [M+H]+ | 814.63 | PC |
| PC(38:3) | [M+H]+ | 812.61 | PC |
| PC(38:4) | [M+H]+ | 810.60 | PC |
| PC(38:5) | [M+H]+ | 808.58 | PC |
| PC(38:6) | [M+H]+ | 806.57 | PC |
| PC(38:6) | [M+K]+ | 844.53 | PC |
| PC(38:7) | [M+H]+ | 804.55 | PC |
| PC(38:7)/PE(41:7) | [M-H]- | 802.54 | PC/PE |
| PC(38:8) | [M+Cl]- | 836.49 | PC |
| PC(39:4) | [M+H]+ | 824.56 | PC |
| PC(39:5) | [M+H]+ | 822.58 | PC |
| PC(39:6) | [M+H]+ | 820.54 | PC |
| PC(39:7) | [M+H]+ | 818.62 | PC |
| PC(40:4) | [M+H]+ | 838.59 | PC |
| PC(40:5) | [M+H]+ | 836.64 | PC |
| PC(40:6) | [M+H]+ | 834.60 | PC |
| PC(40:7) | [M+H]+ | 832.59 | PC |
| PC(40:7) | [M+K]+ | 870.55 | PC |
| PC(40:7) | [M+NH4]+ | 849.60 | PC |
| PC(40:8) | [M+H]+ | 830.56 | PC |
| PC(40:9) | [M+H]+ | 828.55 | PC |
| PC(42:10) | [M+H]+ | 854.56 | PC |
| PC(42:6) | [M+H]+ | 862.70 | PC |
| PC(42:9) | [M+H]+ | 856.61 | PC |
| PC(43:4) | [M+H]+ | 880.78 | PC |
| PC(44:0) | [M+H]+ | 902.82 | PC |
| PE(31:1) | [M-H]- | 674.47 | PE |
| PE(32:1) | [M-H]- | 688.48 | PE |
| PE(33:2) | [M-H]- | 700.48 | PE |
| PE(35:4) | [M-H]- | 724.50 | PE |
| PE(36:1) | [M-H]- | 744.56 | PE |
| **Lipids** | **Adduct** | **m/z** | **Lipid Species** |
| PE(36:2) | [M-H]- | 726.56 | PE |
| PE(36:2) | [M-H]- | 742.54 | PE |
| PE(36:3) | [M-H]- | 740.53 | PE |
| PE(36:4) | [M-H]- | 738.49 | PE |
| PE(36:5) | [M-H]- | 736.49 | PE |
| PE(37:4) | [M-H]- | 752.52 | PE |
| PE(37:5) | [M-H]- | 750.51 | PE |
| PE(37:6) | [M-H]- | 748.48 | PE |
| PE(38:4) | [M-H]- | 766.53 | PE |
| PE(38:6) | [M-H]- | 762.50 | PE |
| PE(38:7) | [M-H]- | 760.48 | PE |
| PE(39:4) | [M-H]- | 780.55 | PE |
| PE(39:5) | [M-H]- | 778.55 | PE |
| PE(39:6) | [M-H]- | 776.52 | PE |
| PE(40:4) | [M-H]- | 778.56 | PE |
| PE(40:6) | [M-H]- | 790.54 | PE |
| PE(40:7) | [M-H]- | 788.51 | PE |
| PE(40:8) | [M-H]- | 786.50 | PE |
| PE(42:10) | [M-H]- | 810.50 | PE |
| PG(33:3) | [M-H]- | 729.49 | PG |
| PG(35:4) | [M-H]- | 755.49 | PG |
| PG(35:5) | [M-H]- | 753.47 | PG |
| PG(36:2) | [M-H]- | 773.52 | PG |
| PG(36:3) | [M-H]- | 771.52 | PG |
| PG(38:2) | [M-H]- | 801.56 | PG |
| PG(38:3) | [M-H]- | 799.54 | PG |
| PG(38:4) | [M-H]- | 797.54 | PG |
| PG(38:5) | [M-H]- | 795.51 | PG |
| PG(40:4) | [M-H]- | 825.56 | PG |
| PG(40:6) | [M-H]- | 821.55 | PG |
| PG(40:7) | [M-H]- | 819.52 | PG |
| PG(40:8) | [M-H]- | 817.51 | PG |
| PG(42:9) | [M-H]- | 843.52 | PG |
| PI(34:1) | [M-H]- | 835.54 | PI |
| PI(34:2) | [M-H]- | 833.52 | PI |
| PI(34:3) | [M-H]- | 831.51 | PI |
| PI(34:5) | [M-H]- | 827.49 | PI |
| PI(36:2) | [M-H]- | 861.56 | PI |
| PI(36:3) | [M-H]- | 859.53 | PI |
| PI(36:4) | [M-H]- | 857.51 | PI |
| PI(37:3) | [M-H]- | 873.55 | PI |
| PI(37:4) | [M-H]- | 871.53 | PI |
| PI(37:5) | [M-H]- | 869.54 | PI |
| PI(37:7) | [M-H]- | 865.48 | PI |
| PI(38:2) | [M-H]- | 889.58 | PI |
| PI(38:3) | [M-H]- | 887.56 | PI |
| PI(38:4) | [M-H]- | 885.55 | PI |
| **Lipids** | **Adduct** | **m/z** | **Lipid Species** |
| PI(38:5) | [M-H]- | 883.54 | PI |
| PI(38:6) | [M-H]- | 881.52 | PI |
| PI(39:4) | [M-H]- | 899.56 | PI |
| PI(40:4) | [M-H]- | 913.58 | PI |
| PI(40:5) | [M-H]- | 911.56 | PI |
| PI(40:6) | [M-H]- | 909.55 | PI |
| PI(42:8) | [M-H]- | 933.55 | PI |
| PI(42:9) | [M-H]- | 931.52 | PI |
| PS(32:0) | [M-H]- | 718.52 | PS |
| PS(33:0) | [M-H]- | 748.50 | PS |
| PS(34:2) | [M-H]- | 758.51 | PS |
| PS(34:4) | [M-H]- | 754.48 | PS |
| PS(35:0) | [M-H]- | 776.54 | PS |
| PS(35:1) | [M-H]- | 774.52 | PS |
| PS(35:3) | [M-H]- | 770.51 | PS |
| PS(36:4) | [M-H]- | 782.51 | PS |
| PS(36:5) | [M-H]- | 780.50 | PS |
| PS(37:3) | [M-H]- | 798.52 | PS |
| PS(37:4) | [M-H]- | 796.50 | PS |
| PS(37:5) | [M-H]- | 794.50 | PS |
| PS(37:6) | [M-H]- | 792.49 | PS |
| PS(38:4) | [M-H]- | 810.53 | PS |
| PS(38:5) | [M-H]- | 808.50 | PS |
| PS(38:6) | [M-H]- | 806.50 | PS |
| PS(38:7) | [M-H]- | 804.50 | PS |
| PS(39:7) | [M-H]- | 818.49 | PS |
| PS(39:8) | [M-H]- | 816.49 | PS |
| PS(40:6) | [M-H]- | 834.53 | PS |
| PS(40:8) | [M-H]- | 830.50 | PS |
| PS(42:10) | [M-H]- | 854.49 | PS |
| PS(42:6) | [M-H]- | 862.56 | PS |
| PS(42:8) | [M-H]- | 858.52 | PS |
| SM(33:1) | [M-H]- | 687.54 | SM |
| SM(34:1) | [M+H]+ | 703.59 | SM |
| SM(36:0) | [M+H]+ | 733.57 | SM |
| SM(38:0) | [M+H]+ | 761.58 | SM |
| SM(38:2) | [M+H]+ | 757.54 | SM |
| SM(40:0) | [M+H]+ | 789.57 | SM |
| SM(40:1) | [M+H]+ | 787.61 | SM |
| SM(40:2) | [M+H]+ | 785.61 | SM |
| SM(40:3) | [M+H]+ | 783.60 | SM |
| SM(41:1) | [M+H]+ | 801.61 | SM |
| SM(41:2) | [M+H]+ | 799.57 | SM |
| SM(42:1) | [M+H]+ | 815.70 | SM |
| SM(42:2) | [M+H]+ | 813.65 | SM |
| SM(42:3) | [M+H]+ | 811.63 | SM |
| SM(43:1) | [M+H]+ | 829.61 | SM |
| **Lipids** | **Adduct** | **m/z** | **Lipid Species** |
| TAG(50:1) | [M+Na]+ | 855.74 | TAG |
| TAG(50:2) | [M+NH4]+ | 848.77 | TAG |
| TAG(50:2) | [M+Na]+ | 853.73 | TAG |
| TAG(50:3) | [M+Na]+ | 851.71 | TAG |
| TAG(50:5) | [M+Na]+ | 847.63 | TAG |
| TAG(51:0) | [M+H]+ | 849.80 | TAG |
| TAG(51:0) | [M+Na]+ | 871.73 | TAG |
| TAG(51:1) | [M+NH4]+ | 864.72 | TAG |
| TAG(51:1) | [M+Na]+ | 869.75 | TAG |
| TAG(51:2) | [M+Na]+ | 867.74 | TAG |
| TAG(51:4) | [M+Na]+ | 863.71 | TAG |
| TAG(51:5) | [M+Na]+ | 861.67 | TAG |
| TAG(52:1) | [M+Na]+ | 883.80 | TAG |
| TAG(52:2) | [M+NH4]+ | 876.83 | TAG |
| TAG(52:2) | [M+Na]+ | 881.79 | TAG |
| TAG(52:3) | [M+NH4]+ | 874.79 | TAG |
| TAG(52:3) | [M+Na]+ | 879.78 | TAG |
| TAG(52:4) | [M+Na]+ | 877.82 | TAG |
| TAG(52:5) | [M+Na]+ | 875.77 | TAG |
| TAG(53:2) | [M+NH4]+ | 890.71 | TAG |
| TAG(53:2) | [M+Na]+ | 895.74 | TAG |
| TAG(53:4) | [M+NH4]+ | 886.72 | TAG |
| TAG(53:4) | [M+Na]+ | 891.75 | TAG |
| TAG(53:5) | [M+Na]+ | 889.73 | TAG |
| TAG(53:6) | [M+Na]+ | 887.72 | TAG |
| TAG(54:0) | [M+NH4]+ | 908.82 | TAG |
| TAG(54:2) | [M+Na]+ | 909.72 | TAG |
| TAG(54:2) | [M+K]+ | 925.81 | TAG |
| TAG(54:3) | [M+H]+ | 885.70 | TAG |
| TAG(54:3) | [M+Na]+ | 907.81 | TAG |
| TAG(54:4) | [M+Na]+ | 905.75 | TAG |
| TAG(54:4) | [M+K]+ | 921.78 | TAG |
| TAG(54:5) | [M+Na]+ | 903.72 | TAG |
| TAG(55:2) | [M+Na]+ | 923.80 | TAG |
| TAG(55:3) | [M+K]+ | 937.74 | TAG |
| TAG(55:4) | [M+NH4]+ | 914.74 | TAG |
| TAG(55:4) | [M+K]+ | 935.79 | TAG |
| TAG(55:6) | [M+NH4]+ | 910.68 | TAG |
| TAG(55:7) | [M+Na]+ | 913.74 | TAG |
| TAG(55:8) | [M+Na]+ | 911.73 | TAG |
| TAG(56:2) | [M+H]+ | 915.79 | TAG |
| TAG(56:6) | [M+NH4]+ | 924.78 | TAG |

| **Table S2: Significant spatial hepatic lipids distributed across the proto-central axis along with the liver zonation of control and MASH tissues using DESI-MSI** | | | | | | | | | | | | | |
| --- | --- | --- | --- | --- | --- | --- | --- | --- | --- | --- | --- | --- | --- |
| **Lipids** | **Chains** | **Lipid Species** | **P VALUE** | **FDR** | **AVE Intensity Central_Control (30 pixels/samples)** | **AVE Intensity Central_MASH (30 pixels/samples)** | **AVE Intensity Portal_Control (30 pixels/samples)** | **AVE Intensity Portal_MASH (30 pixels/samples)** | **log2FC Central** | **log2FC Portal** | **log2FC in predominant zone of MASH** | **Zone Control** | **Zone MASH** |
| Cer(35:0) | Cer(d18:0_17:0) | Cer | 0.0013 | 0.0038 | 16.7000 | 39.4300 | 16.2900 | 47.4100 | 1.2394 | 1.5412 | 1.5412 | Central/Equal | Portal |
| Cer(37:0) | Cer(d18:0_19:0) | Cer | 0.0000 | 0.0000 | 10.9808 | 100.9441 | 8.9149 | 119.4387 | 3.2005 | 3.7439 | 3.7439 | Central | Portal |
| DAG(32:0) | DAG(16:0_16:0) | DAG | 0.0031 | 0.0076 | 4.5100 | 18.5300 | 4.7400 | 22.1900 | 2.0387 | 2.2270 | 2.2270 | Portal | Portal |
| DAG(32:1) | DAG(18:1_14:0) | DAG | 0.0001 | 0.0006 | 3.1400 | 56.4800 | 3.7200 | 69.9200 | 4.1689 | 4.2323 | 4.2323 | Portal | Portal |
| DAG(32:2) |  | DAG | 0.0009 | 0.0029 | 1.8600 | 16.2800 | 2.3400 | 19.4400 | 3.1297 | 3.0544 | 3.0544 | Portal | Portal |
| DAG(33:2) | DAG(15:0_18:2) | DAG | 0.0047 | 0.0111 | 33.8600 | 37.0600 | 30.7900 | 43.6900 | 0.1303 | 0.5048 | 0.5048 | Central | Portal |
| DAG(34:1) | DAG(18:1_16:0) | DAG | 0.0000 | 0.0001 | 31.1500 | 240.8200 | 28.0300 | 306.2900 | 2.9507 | 3.4499 | 3.4499 | Central | Portal |
| DAG(34:2) | DAG(18:1_16:1) | DAG | 0.0005 | 0.0015 | 52.4500 | 126.2800 | 54.5000 | 159.7400 | 1.2676 | 1.5514 | 1.5514 | Portal | Portal |
| DAG(35:3) | DAG(13:0_22:3) | DAG | 0.0016 | 0.0044 | 6.6300 | 30.2000 | 6.6600 | 35.9000 | 2.1875 | 2.4304 | 2.4304 | Portal/Equal | Portal |
| DAG(36:0) | DAG(18:0_18:0) | DAG | 0.0161 | 0.0329 | 6.6800 | 10.6400 | 7.5500 | 10.3400 | 0.6716 | 0.4537 | 0.4537 | Portal | Central/Equal |
| DAG(36:2) | DAG(18:1_18:1) | DAG | 0.0000 | 0.0000 | 25.3800 | 259.7600 | 20.9900 | 324.3100 | 3.3554 | 3.9496 | 3.9496 | Central | Portal |
| DAG(36:3) | DAG(18:1_18:2) | DAG | 0.0008 | 0.0026 | 7.4200 | 17.3400 | 5.9800 | 21.3300 | 1.2246 | 1.8347 | 1.8347 | Central | Portal |
| DAG(38:2) |  | DAG | 0.0002 | 0.0008 | 2.7000 | 14.3000 | 2.4600 | 16.8000 | 2.4050 | 2.7717 | 2.7717 | Central | Portal |
| DAG(38:4) |  | DAG | 0.0017 | 0.0047 | 22.1100 | 22.0800 | 23.7700 | 21.0800 | -0.0020 | -0.1733 | -0.0020 | Portal | Central |
| FA(18:1) (Oleic acid) |  | FA | 0.0001 | 0.0003 | 320.9300 | 2663.6200 | 336.0600 | 3075.9600 | 3.0531 | 3.1942 | 3.1942 | Portal | Portal |
| FA(20:1) |  | FA | 0.0111 | 0.0236 | 6.4100 | 42.6900 | 7.6900 | 50.3900 | 2.7355 | 2.7121 | 2.7121 | Portal | Portal |
| FA(20:4) (Arachidonic acid) |  | FA | 0.0000 | 0.0000 | 101.3400 | 205.0700 | 163.8200 | 212.8700 | 1.0169 | 0.3779 | 0.3779 | Portal | Portal |
| FA(22:3) |  | FA | 0.0030 | 0.0075 | 0.2100 | 5.7200 | 0.7000 | 7.5400 | 4.7676 | 3.4291 | 3.4291 | Portal | Portal |
| FA(22:4) |  | FA | 0.0083 | 0.0182 | 1.8200 | 3.8700 | 5.4200 | 5.6900 | 1.0884 | 0.0701 | 0.0701 | Portal | Portal |
| FA(22:6) (DHA) |  | FA | 0.0000 | 0.0000 | 69.2000 | 70.6300 | 133.9200 | 74.6000 | 0.0295 | -0.8441 | -0.8441 | Portal | Portal |
| LPA(18:0) | LPA(18:0_0:0) | LPL | 0.0000 | 0.0000 | 19.1100 | 5.7200 | 26.4500 | 6.7000 | -1.7402 | -1.9810 | -1.9810 | Portal | Portal |
| LPE(16:0) | LPE(16:0_0:0) | LPL | 0.0000 | 0.0002 | 52.4600 | 40.4500 | 71.7000 | 43.5800 | -0.3751 | -0.7183 | -0.7183 | Portal | Portal |
| PA(37:1) |  | PA | 0.0000 | 0.0000 | 9.5000 | 3.5200 | 16.1600 | 4.3100 | -1.4324 | -1.9067 | -1.9067 | Portal | Portal |
| PA(38:6) |  | PA | 0.0000 | 0.0000 | 7.7300 | 5.1000 | 14.7600 | 6.0200 | -0.6000 | -1.2939 | -1.2939 | Portal | Portal |
| PA(39:1) |  | PA | 0.0001 | 0.0003 | 16.6900 | 5.5500 | 24.5900 | 6.7800 | -1.5884 | -1.8587 | -1.8587 | Portal | Portal |
| PA(40:6) |  | PA | 0.0000 | 0.0000 | 63.9800 | 39.5900 | 143.1400 | 43.8000 | -0.6925 | -1.7084 | -1.7084 | Portal | Portal |
| PA(40:7) | PA(18:1_22:6) | PA | 0.0000 | 0.0000 | 14.5400 | 13.8500 | 26.5300 | 14.9200 | -0.0701 | -0.8304 | -0.8304 | Portal | Portal |
| PA(41:3) | PA(20:3_21:0) | PA | 0.0001 | 0.0005 | 68.0100 | 63.3200 | 87.2200 | 56.1400 | -0.1031 | -0.6356 | -0.1031 | Portal | Central |
| PA(42:6) |  | PA | 0.0017 | 0.0047 | 4.0100 | 6.5900 | 6.8600 | 7.2100 | 0.7167 | 0.0718 | 0.0718 | Portal | Portal |
| PC(28:0) |  | PC | 0.0000 | 0.0000 | 2.2200 | 1.6400 | 4.5100 | 1.9200 | -0.4369 | -1.2320 | -1.2320 | Portal | Portal |
| **Lipids** | **Chains** | **Lipid Species** | **P VALUE** | **FDR** | **AVE Intensity Central_Control (30 pixels)** | **AVE Intensity Central_MASH (30 pixels)** | **AVE Intensity Portal_Control (30 pixels)** | **AVE Intensity Portal_MASH (30 pixels)** | **log2FC Central** | **log2FC Portal** | **log2FC in predominant zone of MASH** | **Zone Control** | **Zone MASH** |
| PC(30:2) | PC(12:0_18:2) | PC | 0.0000 | 0.0000 | 4.0400 | 5.4700 | 8.6500 | 6.6700 | 0.4372 | -0.3750 | -0.3750 | Portal | Portal |
| PC(31:0) | PC(15:0_16:0) | PC | 0.0132 | 0.0275 | 1.1500 | 1.7600 | 2.8300 | 2.5100 | 0.6139 | -0.1731 | -0.1731 | Portal | Portal |
| PC(31:1) |  | PC | 0.0000 | 0.0000 | 4.4800 | 4.1100 | 7.4200 | 4.7500 | -0.1244 | -0.6435 | -0.6435 | Portal | Portal |
| PC(31:2)/PE(34:2) |  | PC/PE | 0.0000 | 0.0000 | 27.2200 | 11.4800 | 48.5900 | 12.5900 | -1.2455 | -1.9484 | -1.9484 | Portal | Portal |
| PC(32:2) | PC(14:0_18:2) | PC | 0.0000 | 0.0000 | 10.4400 | 4.4400 | 18.1900 | 4.9400 | -1.2335 | -1.8806 | -1.8806 | Portal | Portal |
| PC(32:3)/PE(35:3) |  | PC/PE | 0.0000 | 0.0001 | 2.6400 | 2.2600 | 4.4300 | 2.5600 | -0.2242 | -0.7912 | -0.7912 | Portal | Portal |
| PC(33:2) |  | PC | 0.0037 | 0.0089 | 87.9700 | 17.8700 | 107.9400 | 21.9200 | -2.2995 | -2.2999 | -2.2999 | Portal | Portal |
| PC(34:1) | PC(16:0_18:1) | PC | 0.0015 | 0.0042 | 515.7000 | 1117.1500 | 579.0100 | 1034.7000 | 1.1152 | 0.8376 | 1.1152 | Portal | Central |
| PC(34:2) | PC(16:0_18:2) | PC | 0.0001 | 0.0005 | 1712.4900 | 399.9800 | 1797.0300 | 362.1300 | -2.0981 | -2.3110 | -2.0981 | Portal | Central |
| PC(34:3) | PC(16:0_18:3) | PC | 0.0000 | 0.0000 | 119.6400 | 45.0400 | 170.1400 | 47.5600 | -1.4094 | -1.8389 | -1.8389 | Portal | Portal |
| PC(34:4) | PC(12:0_22:4) | PC | 0.0000 | 0.0000 | 14.2300 | 26.6800 | 18.8900 | 25.5600 | 0.9068 | 0.4363 | 0.9068 | Portal | Central |
| PC(35:2) | PC(17:0_18:2) | PC | 0.0201 | 0.0395 | 170.6200 | 27.0700 | 155.5300 | 28.1000 | -2.6560 | -2.4686 | -2.4686 | Central | Portal |
| PC(35:3) | PC(15:0_20:3) | PC | 0.0004 | 0.0014 | 21.2000 | 29.0100 | 27.0100 | 27.6200 | 0.4525 | 0.0322 | 0.4525 | Portal | Central |
| PC(35:4) |  | PC | 0.0004 | 0.0014 | 16.1000 | 22.5800 | 19.6400 | 23.4900 | 0.4880 | 0.2583 | 0.2583 | Portal | Portal |
| PC(35:5) | PC(15:1_20:4) | PC | 0.0000 | 0.0000 | 43.5000 | 225.8400 | 77.4800 | 223.0200 | 2.3762 | 1.5253 | 1.5253 | Portal | Central/Equal |
| PC(36:2) | PC(18:2_18:0) | PC | 0.0002 | 0.0007 | 523.1400 | 312.3400 | 466.8400 | 307.0400 | -0.7441 | -0.6045 | -0.7441 | Central | Central/Equal |
| PC(36:3) | PC(16:0_20:3) | PC | 0.0028 | 0.0072 | 328.3900 | 398.0500 | 338.1900 | 354.7800 | 0.2775 | 0.0691 | 0.2775 | Portal | Central |
| PC(36:5) | PC(16:0_20:5) | PC | 0.0083 | 0.0182 | 358.7800 | 113.0900 | 374.4700 | 99.3200 | -1.6656 | -1.9147 | -1.6656 | Portal | Central |
| PC(36:6) | PC(14:0_22:6) | PC | 0.0000 | 0.0000 | 16.6400 | 13.0700 | 25.1200 | 12.6300 | -0.3484 | -0.9920 | -0.3484 | Portal | Central/equal |
| PC(37:1) | PC(15:0_22:1) | PC | 0.0020 | 0.0053 | 25.8000 | 17.3500 | 23.3300 | 18.2500 | -0.5724 | -0.3543 | -0.3543 | Central | Portal |
| PC(37:2) |  | PC | 0.0000 | 0.0000 | 57.5800 | 23.1000 | 45.7400 | 22.8400 | -1.3177 | -1.0019 | -1.3177 | Central | Central/Equal |
| PC(37:7)/PE(40:7) |  | PC/PE | 0.0002 | 0.0008 | 22.9300 | 75.3200 | 36.9600 | 77.2900 | 1.7158 | 1.0643 | 1.0643 | Portal | Portal |
| PC(38:1) |  | PC | 0.0000 | 0.0000 | 1.9300 | 1.3200 | 4.5400 | 2.0500 | -0.5481 | -1.1471 | -1.1471 | Portal | Portal |
| PC(38:4) | PC(18:0_20:4) | PC | 0.0191 | 0.0385 | 263.6900 | 252.5500 | 220.7300 | 235.6900 | -0.0623 | 0.0946 | -0.0623 | Central | Central |
| PC(38:6) | PC(16:0_22:6) | PC | 0.0011 | 0.0033 | 556.1700 | 332.6600 | 592.9600 | 280.7800 | -0.7415 | -1.0785 | -0.7415 | Portal | Central |
| PC(39:7) | PC(17:1_22:6) | PC | 0.0019 | 0.0051 | 25.7200 | 8.2100 | 21.9800 | 8.7200 | -1.6474 | -1.3338 | -1.3338 | Central | Portal |
| PC(40:4) | PC(18:0_22:4) | PC | 0.0000 | 0.0002 | 35.0400 | 11.5800 | 30.8000 | 12.4100 | -1.5974 | -1.3114 | -1.3114 | Central | Portal |
| PC(42:10) |  | PC | 0.0027 | 0.0068 | 41.0700 | 15.7400 | 35.7700 | 14.4100 | -1.3836 | -1.3117 | -1.3836 | Central | Central |
| PC(42:6) | PC(20:0_22:6) | PC | 0.0023 | 0.0061 | 25.2092 | 24.4573 | 31.6070 | 23.1014 | -0.0437 | -0.4523 | -0.0437 | Portal | Central |
| PC(42:9) |  | PC | 0.0000 | 0.0000 | 30.3000 | 12.3400 | 23.2700 | 11.2000 | -1.2960 | -1.0550 | -1.2960 | Central | Central |
| **Lipids** | **Chains** | **Lipid Species** | **P VALUE** | **FDR** | **AVE Intensity Central_Control (30 pixels)** | **AVE Intensity Central_MASH (30 pixels)** | **AVE Intensity Portal_Control (30 pixels)** | **AVE Intensity Portal_MASH (30 pixels)** | **log2FC Central** | **log2FC Portal** | **log2FC in predominant zone of MASH** | **Zone Control** | **Zone MASH** |
| PC(43:4) | PC(21:0_22:4) | PC | 0.0193 | 0.0385 | 3.3200 | 17.3900 | 2.6900 | 23.2000 | 2.3890 | 3.1084 | 3.1084 | Central | Portal |
| PC(44:0) | PC(18:0_26:0) | PC | 0.0012 | 0.0036 | 1.9200 | 21.4800 | 1.4600 | 29.4500 | 3.4838 | 4.3342 | 4.3342 | Central | Portal |
| PE(33:2) |  | PE | 0.0000 | 0.0000 | 2.6000 | 2.1300 | 6.0600 | 2.8600 | -0.2877 | -1.0833 | -1.0833 | Portal | Portal |
| PE(36:3) | PE(18:1_18:2) | PE | 0.0000 | 0.0000 | 16.0500 | 23.0600 | 29.4500 | 24.2000 | 0.5228 | -0.2833 | 0.5228 | Portal | Portal |
| PE(36:4) | PE(16:0_20:4) | PE | 0.0000 | 0.0000 | 27.6500 | 102.9700 | 51.4900 | 102.2500 | 1.8969 | 0.9897 | 1.8969 | Portal | Central/Equal |
| PE(36:5) |  | PE | 0.0000 | 0.0000 | 3.9800 | 8.0800 | 7.8200 | 9.2100 | 1.0216 | 0.2360 | 0.2360 | Portal | Portal |
| PE(37:4) |  | PE | 0.0103 | 0.0223 | 3.2700 | 4.5700 | 5.8700 | 5.9400 | 0.4829 | 0.0171 | 0.0171 | Portal | Portal |
| PE(37:6) |  | PE | 0.0000 | 0.0000 | 16.4400 | 9.0200 | 35.5400 | 11.0200 | -0.8660 | -1.6893 | -1.6893 | Portal | Portal |
| PE(38:4) | PE(15:0_20:4) | PE | 0.0000 | 0.0002 | 271.7800 | 286.3900 | 361.5200 | 263.2000 | 0.0755 | -0.4579 | -0.4579 | Portal | Central |
| PE(38:6) | PE(16:0_22:6) | PE | 0.0000 | 0.0000 | 126.4700 | 347.4700 | 300.0600 | 333.4900 | 1.4581 | 0.1524 | 1.4581 | Portal | Central |
| PE(38:7) | PE(16:1_22:6 | PE | 0.0002 | 0.0008 | 3.6400 | 5.3200 | 7.4400 | 6.3400 | 0.5475 | -0.2308 | -0.2308 | Portal | Portal |
| PE(39:5) | PE(16:1_20:4) | PE | 0.0061 | 0.0140 | 2.2300 | 3.6700 | 3.9700 | 4.2100 | 0.7187 | 0.0847 | 0.0847 | Portal | Portal |
| PE(39:6) | PE(17:0_22:6) | PE | 0.0000 | 0.0000 | 4.9400 | 6.0500 | 10.1300 | 7.0900 | 0.2924 | -0.5148 | -0.5148 | Portal | Portal |
| PE(40:6) | PE(22:6_18:0) | PE | 0.0000 | 0.0000 | 66.9300 | 53.3900 | 98.9000 | 54.4600 | -0.3261 | -0.8608 | -0.8608 | Portal | Portal/Equal |
| PE(40:8) |  | PE | 0.0002 | 0.0006 | 7.1300 | 4.2200 | 10.3800 | 4.9500 | -0.7567 | -1.0683 | -1.0683 | Portal | Portal |
| PE(42:10) | PE(20:4_22:6) | PE | 0.0149 | 0.0309 | 35.6500 | 14.3700 | 31.7200 | 14.8400 | -1.3108 | -1.0959 | -1.0959 | Central | Portal/Equal |
| PG(33:3) |  | PG | 0.0000 | 0.0000 | 3.6500 | 3.0200 | 7.9500 | 3.3400 | -0.2733 | -1.2511 | -1.2511 | Portal | Portal |
| PG(35:4) |  | PG | 0.0001 | 0.0002 | 2.9800 | 2.7200 | 5.2200 | 3.0800 | -0.1317 | -0.7611 | -0.7611 | Portal | Portal |
| PG(35:5) |  | PG | 0.0073 | 0.0163 | 3.4500 | 2.2600 | 5.0600 | 2.4600 | -0.6103 | -1.0405 | -1.0405 | Portal | Portal |
| PG(36:3) |  | PG | 0.0000 | 0.0001 | 7.7700 | 14.1300 | 12.0400 | 14.2300 | 0.8628 | 0.2411 | 0.2411 | Portal | Portal/Equal |
| PG(40:7) | PC(18:1_22:6) | PG | 0.0001 | 0.0005 | 7.2000 | 20.4100 | 10.1500 | 17.8800 | 1.5032 | 0.8169 | 1.5032 | Portal | Central |
| PG(40:8) |  | PG | 0.0066 | 0.0151 | 6.9300 | 3.1700 | 9.1500 | 3.5500 | -1.1284 | -1.3660 | -1.3660 | Portal | Portal |
| PI(34:1) |  | PI | 0.0000 | 0.0000 | 9.6100 | 5.4800 | 17.3700 | 6.6000 | -0.8104 | -1.3961 | -1.3961 | Portal | Portal |
| PI(34:2) |  | PI | 0.0004 | 0.0014 | 33.6500 | 3.9200 | 46.8200 | 4.2100 | -3.1017 | -3.4752 | -3.4752 | Portal | Portal |
| PI(36:2) | PI(18:2_18:0) | PI | 0.0000 | 0.0000 | 30.3900 | 14.6200 | 51.2900 | 15.9600 | -1.0557 | -1.6842 | -1.6842 | Portal | Portal |
| PI(36:3) |  | PI | 0.0024 | 0.0062 | 30.7800 | 68.4000 | 48.9600 | 65.8600 | 1.1520 | 0.4278 | 1.1520 | Portal | Central |
| PI(36:4) |  | PI | 0.0000 | 0.0000 | 86.9700 | 63.9500 | 135.9800 | 61.9300 | -0.4436 | -1.1347 | -0.4436 | Portal | Central/equal |
| PI(37:3) | PI(17:0_20:3) | PI | 0.0000 | 0.0000 | 2.3200 | 8.8400 | 3.5900 | 8.0700 | 1.9299 | 1.1686 | 1.9299 | Portal | Central |
| PI(37:4) |  | PI | 0.0002 | 0.0008 | 6.0000 | 4.6000 | 8.1000 | 4.6800 | -0.3833 | -0.7914 | -0.7914 | Portal | Portal |
| PI(37:7) |  | PI | 0.0000 | 0.0001 | 15.0000 | 2.2400 | 19.8900 | 2.3300 | -2.7434 | -3.0936 | -3.0936 | Portal | Portal |
| **Lipids** | **Chains** | **Lipid Species** | **P VALUE** | **FDR** | **AVE Intensity Central_Control (30 pixels)** | **AVE Intensity Central_MASH (30 pixels)** | **AVE Intensity Portal_Control (30 pixels)** | **AVE Intensity Portal_MASH (30 pixels)** | **log2FC Central** | **log2FC Portal** | **log2FC in predominant zone of MASH** | **Zone Control** | **Zone MASH** |
| PI(38:2) | PI(18:0_20:2) | PI | 0.0000 | 0.0000 | 9.0700 | 80.9300 | 9.6300 | 63.1800 | 3.1575 | 2.7139 | 3.1575 | Portal | Central |
| PI(38:4) | PI(18:0_20:4) | PI | 0.0000 | 0.0000 | 5826.0500 | 2477.9800 | 4863.1800 | 2202.3800 | -1.2334 | -1.1428 | -1.2334 | Central | Central |
| PI(38:5) | PI(18:1_20:4) | PI | 0.0038 | 0.0091 | 34.3200 | 191.0700 | 53.8900 | 168.4200 | 2.4770 | 1.6440 | 2.4770 | Portal | Central |
| PI(38:6) |  | PI | 0.0000 | 0.0002 | 5.7000 | 2.5500 | 8.0100 | 2.8300 | -1.1605 | -1.5010 | -1.5010 | Portal | Portal |
| PI(39:4) | PI(17:0_22:4) | PI | 0.0000 | 0.0000 | 19.2500 | 4.2700 | 15.0900 | 4.4300 | -2.1726 | -1.7682 | -1.7682 | Central | Portal |
| PI(40:5) |  | PI | 0.0208 | 0.0406 | 4.2700 | 3.6700 | 5.5800 | 3.6900 | -0.2185 | -0.5966 | -0.5966 | Portal | Portal/Equal |
| PI(42:8) | PI(20:2_22:6) | PI | 0.0000 | 0.0000 | 20.7200 | 2.2900 | 11.9300 | 2.6600 | -3.1776 | -2.1651 | -2.1651 | Central | Portal |
| PI(42:9) | PI(20:3_22:6) | PI | 0.0000 | 0.0000 | 57.9900 | 2.9000 | 26.0800 | 3.0300 | -4.3217 | -3.1056 | -3.1056 | Central | Portal |
| PS(32:0) | PS(16:0_16:0) | PS | 0.0000 | 0.0001 | 1.0800 | 2.0000 | 3.1600 | 2.6000 | 0.8890 | -0.2814 | -0.2814 | Portal | Portal |
| PS(33:0) |  | PS | 0.0000 | 0.0000 | 22.5600 | 18.8000 | 49.9900 | 23.5800 | -0.2630 | -1.0841 | -1.0841 | Portal | Portal |
| PS(34:2) |  | PS | 0.0110 | 0.0235 | 5.3400 | 2.2800 | 7.9500 | 3.1400 | -1.2278 | -1.3402 | -1.3402 | Portal | Portal |
| PS(34:4) |  | PS | 0.0012 | 0.0034 | 7.0500 | 4.7000 | 11.0200 | 5.7800 | -0.5850 | -0.9310 | -0.9310 | Portal | Portal |
| PS(35:0) |  | PS | 0.0061 | 0.0140 | 2.5000 | 4.5300 | 5.4500 | 5.9000 | 0.8576 | 0.1145 | 0.1145 | Portal | Portal |
| PS(36:5) |  | PS | 0.0195 | 0.0387 | 17.7200 | 10.9400 | 20.9400 | 11.9600 | -0.6958 | -0.8080 | -0.8080 | Portal | Portal |
| PS(37:6) |  | PS | 0.0008 | 0.0026 | 15.1100 | 7.8200 | 18.7400 | 8.3300 | -0.9503 | -1.1697 | -1.1697 | Portal | Portal |
| PS(38:6) |  | PS | 0.0000 | 0.0001 | 11.6100 | 5.2000 | 16.1100 | 5.7000 | -1.1588 | -1.4989 | -1.4989 | Portal | Portal |
| PS(38:7) |  | PS | 0.0016 | 0.0044 | 14.4900 | 6.1100 | 19.7500 | 7.0800 | -1.2458 | -1.4800 | -1.4800 | Portal | Portal |
| PS(40:6) |  | PS | 0.0000 | 0.0000 | 30.3800 | 5.8800 | 47.6400 | 6.7500 | -2.3692 | -2.8192 | -2.8192 | Portal | Portal |
| PS(40:8) | PS(18:2_22:6) | PS | 0.0179 | 0.0365 | 16.9300 | 3.2200 | 14.0700 | 3.2500 | -2.3944 | -2.1141 | -2.1141 | Central | Portal |
| PS(42:6) |  | PS | 0.0000 | 0.0000 | 13.1400 | 5.1400 | 22.6500 | 5.7500 | -1.3541 | -1.9779 | -1.9779 | Portal | Portal |
| PS(42:8) | PS(20:4_22:4) | PS | 0.0000 | 0.0000 | 40.2000 | 25.9500 | 57.2000 | 25.9100 | -0.6315 | -1.1425 | -0.6315 | Portal | Central/Equal |
| SM(34:1) | SM(d16:1_18:0) | SM | 0.0056 | 0.0131 | 13.8400 | 21.8900 | 19.7600 | 35.1400 | 0.6614 | 0.8305 | 0.8305 | Portal | Portal |
| SM(38:0) | SM(d16:0_22:0) | SM | 0.0071 | 0.0160 | 163.4500 | 459.2200 | 188.0600 | 432.9700 | 1.4903 | 1.2031 | 1.4903 | Portal | Central |
| SM(38:2) | SM(d16:1_22:1) | SM | 0.0000 | 0.0000 | 54.7572 | 23.5934 | 76.0517 | 25.6453 | -1.2147 | -1.5683 | -1.5683 | Portal | Portal |
| SM(40:1) | SM(d16:1_24:0) | SM | 0.0000 | 0.0000 | 233.7300 | 131.5200 | 203.1400 | 136.9200 | -0.8296 | -0.5691 | -0.5691 | Central | Portal |
| SM(41:1) | SM(d16:1_25:0) | SM | 0.0000 | 0.0000 | 27.0500 | 10.9300 | 22.0300 | 11.6300 | -1.3073 | -0.9216 | -0.9216 | Central | Portal |
| SM(42:1) | SM(d18:0_24:1) | SM | 0.0010 | 0.0032 | 18.6100 | 14.1300 | 19.1000 | 17.6000 | -0.3973 | -0.1180 | -0.1180 | Portal/Equal | Portal |
| SM(42:2) | SM(d18:1_24:1) | SM | 0.0003 | 0.0011 | 34.2000 | 56.2300 | 33.9600 | 64.2500 | 0.7173 | 0.9199 | 0.9199 | Central/Equal | Portal |
| SM(42:3) | SM(d18:2_24:1) | SM | 0.0009 | 0.0030 | 101.2100 | 91.2700 | 83.7500 | 88.3500 | -0.1491 | 0.0771 | -0.1491 | Central | Central |
| TAG(50:2) | TAG(18:2_16:0_16:0) | TAG | 0.0003 | 0.0009 | 1.3700 | 20.9700 | 1.3700 | 28.1500 | 3.9361 | 4.3609 | 4.3609 | Equal | Portal |
| **Lipids** | **Chains** | **Lipid Species** | **PVALUE** | **FDR** | **AVE Intensity Central_Control (30 pixels)** | **AVE Intensity Central_MASH (30 pixels)** | **AVE Intensity Portal_Control (30 pixels)** | **AVE Intensity Portal_MASH (30 pixels)** | **log2FC Central** | **log2FC Portal** | **log2FC in predominant zone of MASH** | **Zone Control** | **Zone MASH** |
| TAG(50:3) | TAG(20:2_16:1_14:0) | TAG | 0.0234 | 0.0450 | 3.8400 | 14.1100 | 4.1100 | 18.0100 | 1.8775 | 2.1316 | 2.1316 | Portal | Portal |
| TAG(50:5) | TAG(12:0_18:4_20:1) | TAG | 0.0030 | 0.0074 | 10.8400 | 6.6000 | 8.6300 | 6.0300 | -0.7158 | -0.5172 | -0.7158 | Central | Central |
| TAG(51:0) | TAG(12:0_19:0_20:0) | TAG | 0.0003 | 0.0009 | 6.6389 | 10.4252 | 5.7244 | 13.3043 | 0.6510 | 1.2167 | 1.2167 | Central | Portal |
| TAG(51:2) | TAG(18:1_18:1_15:0) | TAG | 0.0234 | 0.0450 | 2.9400 | 8.3300 | 2.8900 | 10.9400 | 1.5025 | 1.9205 | 1.9205 | Central/Equal | Portal |
| TAG(51:5) | TAG(12:0_17:0_22:5) | TAG | 0.0023 | 0.0061 | 60.2600 | 37.4800 | 77.0300 | 36.2100 | -0.6851 | -1.0890 | -0.6851 | Portal | Central |
| TAG(52:2) | TAG(18:0_18:1_16:1) | TAG | 0.0013 | 0.0037 | 3.1500 | 32.4900 | 2.1100 | 45.3100 | 3.3666 | 4.4245 | 4.4245 | Central | Portal |
| TAG(52:3) | TAG(18:1_18:1_16:1) | TAG | 0.0004 | 0.0012 | 10.6100 | 26.9200 | 9.3700 | 36.7200 | 1.3433 | 1.9704 | 1.9704 | Central | Portal |
| TAG(52:4) | TAG(18:2_18:2_16:0) | TAG | 0.0011 | 0.0032 | 1.9300 | 21.8100 | 1.3400 | 28.8500 | 3.4983 | 4.4283 | 4.4283 | Central | Portal |
| TAG(52:5) | TAG(18:2_18:2_16:1) | TAG | 0.0004 | 0.0012 | 6.4436 | 15.9320 | 5.5865 | 21.1153 | 1.3060 | 1.9183 | 1.9183 | Central | Portal |
| TAG(54:4) | TAG(18:1_18:1_18:2) | TAG | 0.0115 | 0.0242 | 3.4500 | 12.1000 | 3.1700 | 16.1400 | 1.8103 | 2.3481 | 2.3481 | Central | Portal |
